# CT-scan based anatomical study as a guidance for infra-acetabular screw placement

**DOI:** 10.1186/s12891-021-04419-x

**Published:** 2021-06-24

**Authors:** Quanyi Lu, Runtao Zhou, Shichang Gao, Anlin Liang, Mingming Yang, Haitao Yang

**Affiliations:** 1 Department of Orthopedics, the First Affiliated Hospital of Chongqing Medical University, Chongqing, 400016 China; 2 Department of Radiology, the First Affiliated Hospital of Chongqing Medical University, Chongqing, 400016 China

**Keywords:** Acetabular fracture, Infra-acetabular corridor, Computed tomography measurement, All-in screw, In-out-in screw

## Abstract

**Background:**

The infra-acetabular corridor is quite narrow, which makes a challenge for the orthopedists to insert the screw. This study aimed to explore the relationship between the infra-acetabular corridor diameter (IACD) and the minimum thickness of medial acetabular wall (MTMAW), and to clarify the way of screw placement.

**Methods:**

The Computed tomography (CT) data of 100 normal adult pelvises (50 males and 50 females respectively) were collected and pelvis three-dimensional (3D) reconstruction was performed by using Mimics software and the 3D model was imported into Geomagic Studio software. The perspective of acetabulum was carried out orienting from iliopubic eminence to ischial tuberosity and the IACD was measured by placing virtual screws which was vertical to the corridor transverse section of “teardrop”. The relationship between IACD and MTMAW was analyzed. When IACD was ≥5 mm, 3.5 mm all-in screws were placed. When IACD was < 5 mm, 3.5 mm in-out-in screws were placed.

**Results:**

The IACD of males and females were (6.15 ± 1.24) mm and (5.42 ± 1.01) mm and the MTMAW in males and females were (4.40 ± 1.23) mm and (3.60 ± 0.81) mm respectively. The IACD and MTMAW in males were significantly wider than those of females (*P* < 0.05), and IACD was positively correlated with MTMAW (*r* = 0.859), the regression equation was IACD = 2.111 + 0.917 MTMAW. In the all-in screw group, 38 cases (76%) were males and 33 cases (66%) were females respectively. The entry point was located at posteromedial of the apex of iliopubic eminence, and the posterior distance and medial distance were (8.03 ± 2.01) mm and (8.49 ± 2.68) mm respectively in males. As for females, those were (8.68 ± 2.35) mm and (8.87 ± 2.79) mm respectively. In the in-out-in screw group, 12 cases (24%) were males and 17 cases (34%) were females, respectively. The posterior distance and medial distance between the entry point and the apex of iliopubic eminence were (10.49 ± 2.58) mm and (6.17 ± 1.84) mm respectively in males. As for females, those were (10.10 ± 2.63) mm and (6.63 ± 1.49) mm respectively. The angle between the infra-acetabular screw and the sagittal plane was medial inclination (0.42 ± 6.49) °in males, lateral inclination (8.09 ± 6.33) °in females, and the angle between the infra-acetabular screw and the coronal plane was posterior inclination (54.06 ± 7.37) °.

**Conclusions:**

The placement mode of the infra-acetabular screw (IAS) can be determined preoperatively by measuring the MTMAW in the CT axial layers. Compared with all-in screw, the in-out-in screw entry point was around 2 mm outwards and backwards, and closer to true pelvic rim.

## Introduction

Acetabular fractures are intra-articular fractures. As for those fractures, open reduction and internal fixation has been the gold standard to achieve anatomic or near-anatomic reduction of the articular surface, avoid complications of recumbency and return to pre-injury function as quickly as possible [1, 2]. Periacetabular fixation frame has been introduced by Culemann [3] for the anterior column fractures. Infra-acetabular screw closes the incomplete periacetabular fixation frame which consists of both osseous columns, the ilioinguinal plate, and supra-acetabular screw fixation. The entry point for the screw is 1 cm caudal of the eminentia iliopectinea in the mid-width of the pubic ramus and the target point is in ischial tuberosity. Infra-acetabular screw placed strictly parallel to the quadrilateral plate surface transfixes both columns, which significantly increases the fixation strength of a standard plate fixation for anterior column fractures. The indications of Infra-acetabular screw include anterior column, anterior column and posterior hemitransverse, T-type and both column fractures. The 3.5 mm screws and reconstruction plates are commonly used for acetabular fractures, and the screw perpendicular to the fracture line is more effective and less traumatic [4, 5]. Gras [6] reported that an infra-acetabular corridor (IAC) with a diameter of at least 5 mm was found in 93% of pelvises. The placement of a 3.5-mm cortical screw through the corridor was achievable. However, another study showed that over 20% of infra-acetabular corridors were not feasible for infra-acetabular screw placement even with the perfect reduction of fragments when treating acetabular fractures [7]. The corridor is adjoining with obturator never, obturator vessels and hip joint and the corridor size exists variance in races and sex, which could cause iatrogenic injury when operation is not proper [8].

Therefore, the present study using the Mimics software and Geomagic Studio software simulated the placement of infra-acetabular screw, measured the infra-acetabular corridor diameter and thickness of medial acetabular wall, clarified the entry point, orientation, safety range and length of infra-acetabular screw, analyzed the relationship between infra-acetabular corridor diameter and minimum thickness of medial acetabular wall based on CT data to provide reference values for the placement of infra-acetabular screw.

## Materials and methods

### Inclusion and exclusion criteria

Inclusion criteria:

1. Pelvis and acetabulum were included in the Computed tomography examination.
2. Pelvis, acetabulum, and sacroiliac joint had no pathological changes.
3. Participator had no compulsive position while being scanned.

Exclusion criteria:

1. Participators with tumor, fracture, or infection.
2. Deformity of pelvis.
3. Participator maintained compulsive position while being scanned, caused by acute abdominal diseases or other emergent diseases.

### Data collection

The CT data of pelvis were collected from the department of radiology in the First Affiliated Hospital of Chongqing Medical University. All procedures performed in studies were in accordance with the 1964 Helsinki declaration. From October 2018 to October 2019, 100 patients (50 women, 50 men) enrolled in the present study, aged from 18 to 86 years old (mean age was 48.96 ± 18.48). The data were reserved by digital imaging communication in medicine (DICOM) format. Scanning parameters: tube voltage was 130 kV, tube current was 100 mA, layer thickness was 0.625 mm and scanning matrix was 512 × 512.

### Semi-pelvic model building

The DICOM format data were imported into Mimics 19.0 software (Materialise, Belgium) and 3D reconstruction of the pelvis was performed. The reconstructed 3D model of the pelvis was imported into the Geomagic Studio 2015 software (Geomagic, USA) in stereolithography (STL) format. According to the method proposed by Feng et al. [9], the triangular facets in the acetabular medullary cavity were removed to make the medullary cavity hollow. The processed model was imported into Mimics 19.0 software in STL format to match the original model.

### Measurement of IACD and grouping

**Fig. 1:**
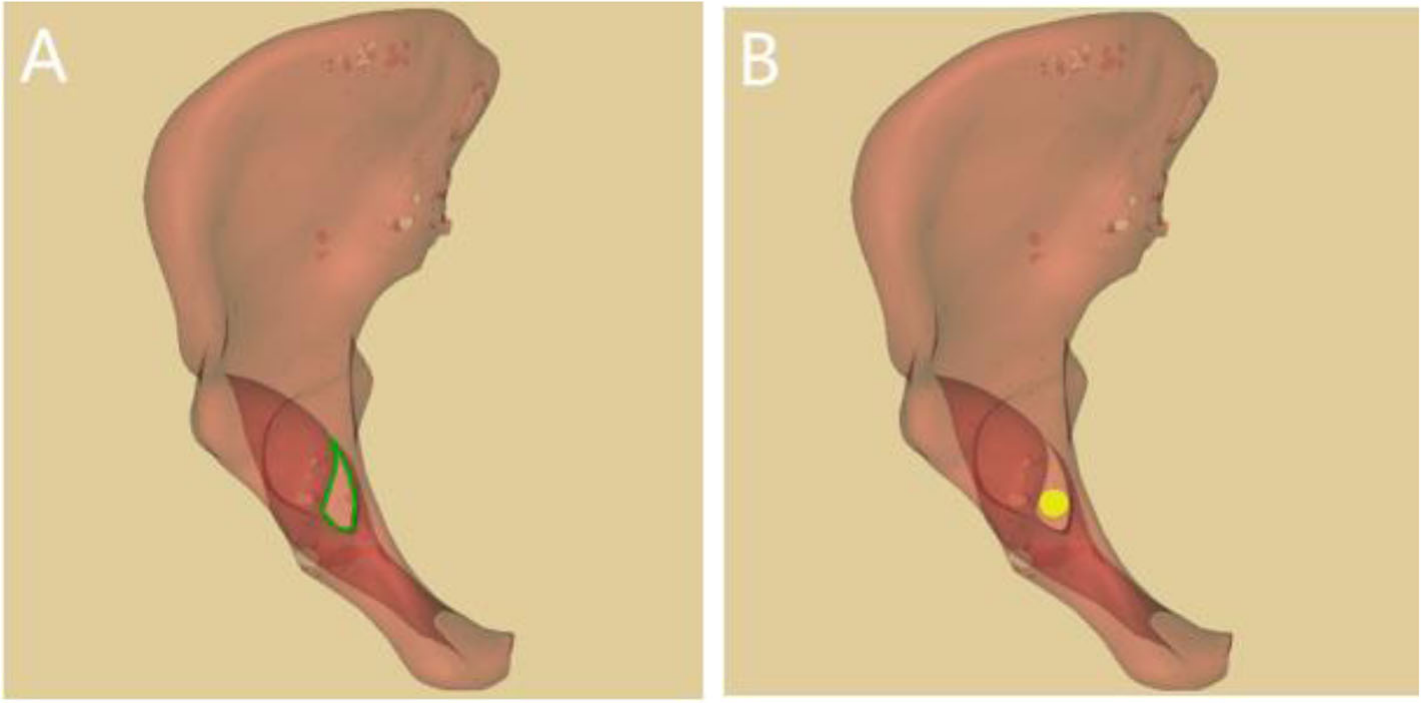
Insertion of virtual screw. 1A: Axial perspective view of infra-acetabular corridor (IAC) and the green teardrop is the safe zone for screw placement. 1B: Axial perspective view of all-in screw

The prepared pelvis model was rotated to inlet view, then the perspective of acetabulum was carried out orienting from iliopubic eminence to ischial tuberosity in the Mimics software. The teardrop area encircled by the medial acetabular wall, the quadrilateral plate, and the posterior of obturator was demonstrated. We adjusted the pelvic model to make the medial of the ischial tuberosity and the roof of the acetabular fossa just overlap. Whereafter, the virtual screw was inserted perpendicular to the cross section of teardrop area. The maximum IACD [9] was achieved by gradually increasing the screw diameter when the screw just did not penetrate the cortex (Fig. 1). We defined the all-in screw as the full length of the screw was in the infra-acetabular corridor and the screw did not penetrate the cortex. Correspondingly, the in-out-in screw was defined as 1/2 screw diameter exposed out of the cortex of quadrilateral plate. When IACD was ≥5.0 mm, a 3.5 mm diameter all-in screw (all-in screw group) was virtually inserted, and when IACD was < 5.0 mm, a 3.5 mm diameter in-out-in screw was inserted (in-out-in screw group).

### Measurement of MTMAW

**Fig. 2:**
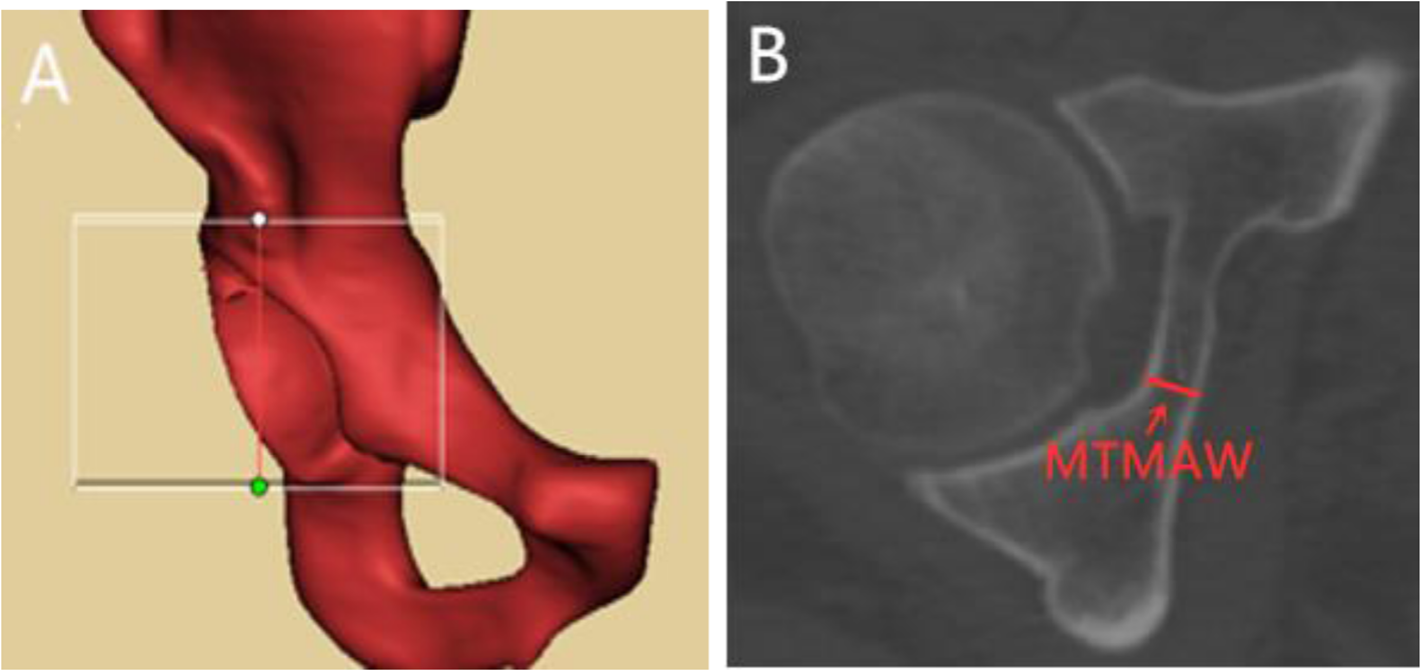
Measurement of MTMAW. 2A: Re-cut range was from the superior of the acetabulum to the inferior of the acetabulum. 2B: Axial view of acetabulum, MTMAW means the minimum thickness of the medial acetabular wall

We used the re-cut function of the Mimics software to cut the acetabulum from the superior to the inferior of the acetabulum with an interval of 1 mm, which was perpendicular to the longitudinal axis of the human body. The thickness of the medial acetabular wall was measured in every layer to obtain the MTMAW (Fig. 2), then the distance between the superior of the acetabulum and the layer of MTMAW was determined.

### Parameter measurement of all-in screw

**Fig. 3:**
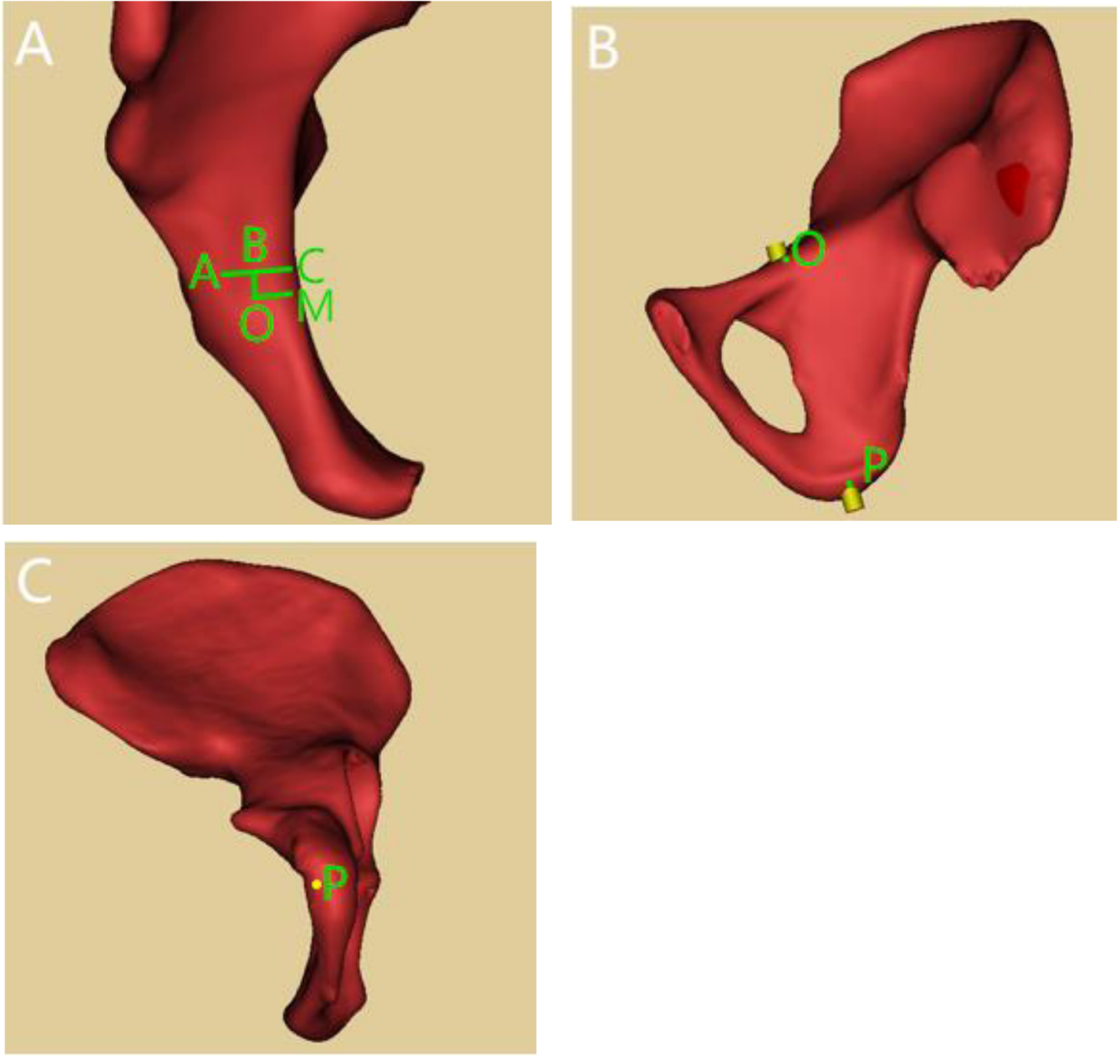
Parameter measurement of all-in screw. 3A: Point A: apex of iliopubic eminence; Point O: entry point; Line segment AC: perpendicular line of true pelvic rim; Line segment AB: posterior distance between the entry point and the apex of iliopubic eminence; Line segment OB: medial distance between the entry point and the apex of iliopubic eminence/ 3B: Lateral view of right hemipelvis; Point P: exit point of all-in screw at the ischial tuberosity. 3C: Bottom view of right hemipelvis; Point P: exit point of all-in screw at the ischial tuberosity

When IACD was ≥5.0 mm, an all-in screw was virtually inserted. We reduced the screw diameter to 0.5 mm, then the screw and the superior ramus of pubis intersected at point O (the entry point). Passing the apex of iliopubic eminence (point A), we drawn the perpendicular line of true pelvic rim and obtained the intersection C. Passing point O, the perpendicular line of line AC was drawn and intersecting Point B. We obtained the posterior distance and medial distance between the entry point and the apex of iliopubic eminence by measuring the length of line segments AB and OB. The line which passed point O was perpendicular to the true pelvic and intersected point M. The distance between entry point and true pelvic rim was got by measuring the length of line segment OM. The screw and the ischial tuberosity intersected at point P (the exit point), and line segment OP was the length of the IAC (Fig. 3).

### Parameter measurement of in-out-in screw

**Fig. 4:**
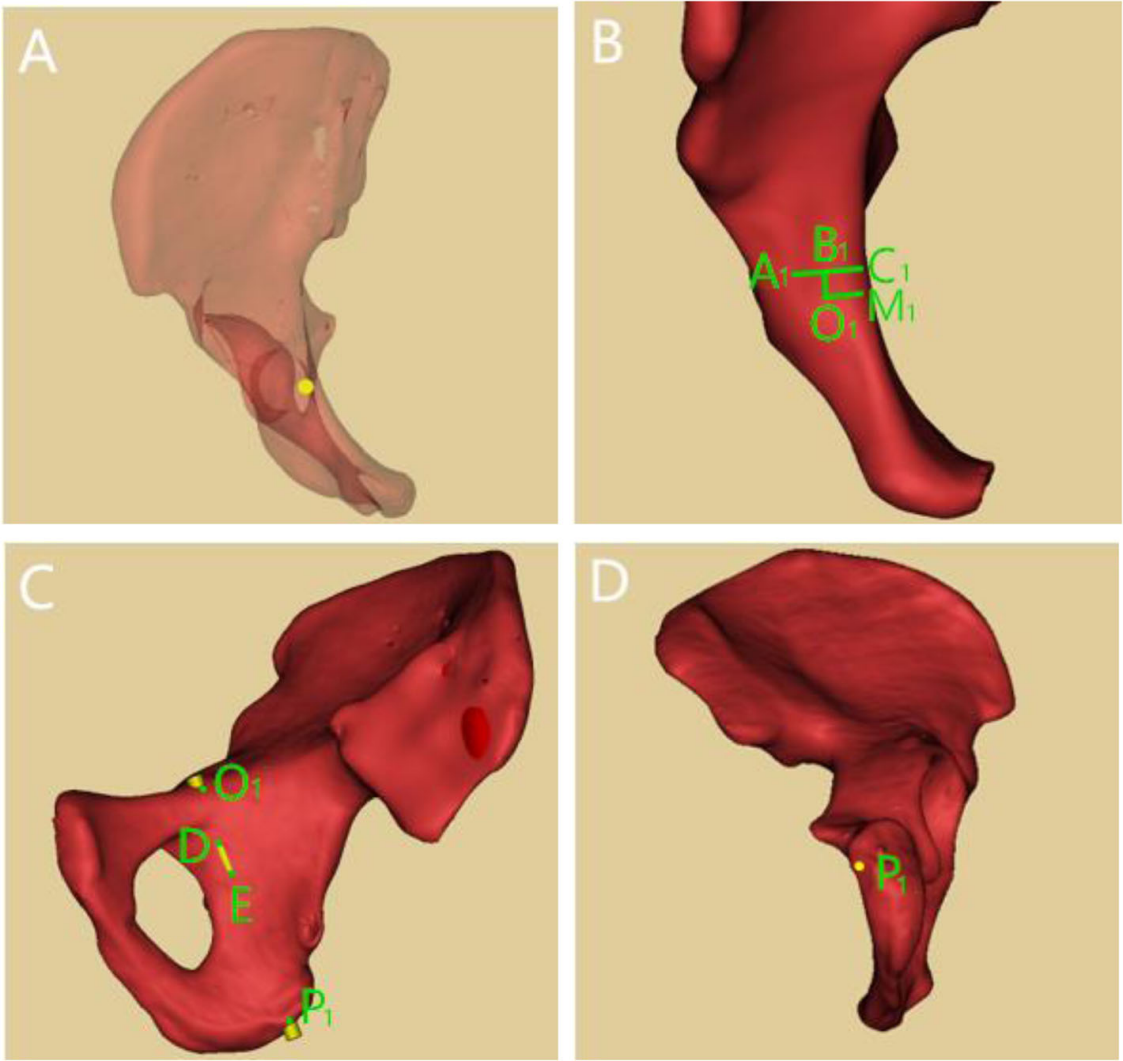
Parameter measurement of in-out-in screw. 4A: Axial perspective view of in-out-in screw. 4B: Point A_1_: apex of iliopubic eminence; Point O_1_: entry point; Line segment A_1_C_1_: perpendicular line of pelvic rim; Line segment A_1_B_1_: posterior distance between the entry point and the apex of iliopubic eminence; Line segment O_1_B_1_: medial distance between the entry point and the apex of iliopubic eminence. 4C: Lateral view of pelvis; Point P_1_: exit point of in-out-in screw at the ischial tuberosity; Point D: proximal perforation in the quadrilateral plate; Point E: distal perforation in the quadrilateral plate. 4D: Bottom view of right hemipelvis; Point P_1_: exit point of in-out-in screw in the ischial tuberosity

When IACD was < 5.0 mm, we moved the entry point of 3.5 mm virtual screw outwards and backwards, and the screw penetrated cortex at the lateral of obturator groove, allowing the 1/2 diameter of the screw exposed out of the cortex of the quadrilateral plate. Point D was proximal perforation in the quadrilateral plate and the point E was the distal perforation, therefore line segment DE represented the length of screw outside of the cortex. When the screw diameter was reduced to 0.5 mm, the screw intersected the superior ramus of pubis at point O_1_(the entry point), and intersected the ischial tuberosity at point P_1_(the exit point). In the same manner previously, we obtained the medial distance and posterior distance between the entry point and the apex of iliopubic eminence by measuring the length of line segments A_1_B_1_ and O_1_B_1_, and the line segment O_1_P_1_ was the length of the IAC (Fig. 4).

### Calculation of virtual screw entry angle and safety range

**Fig. 5:**
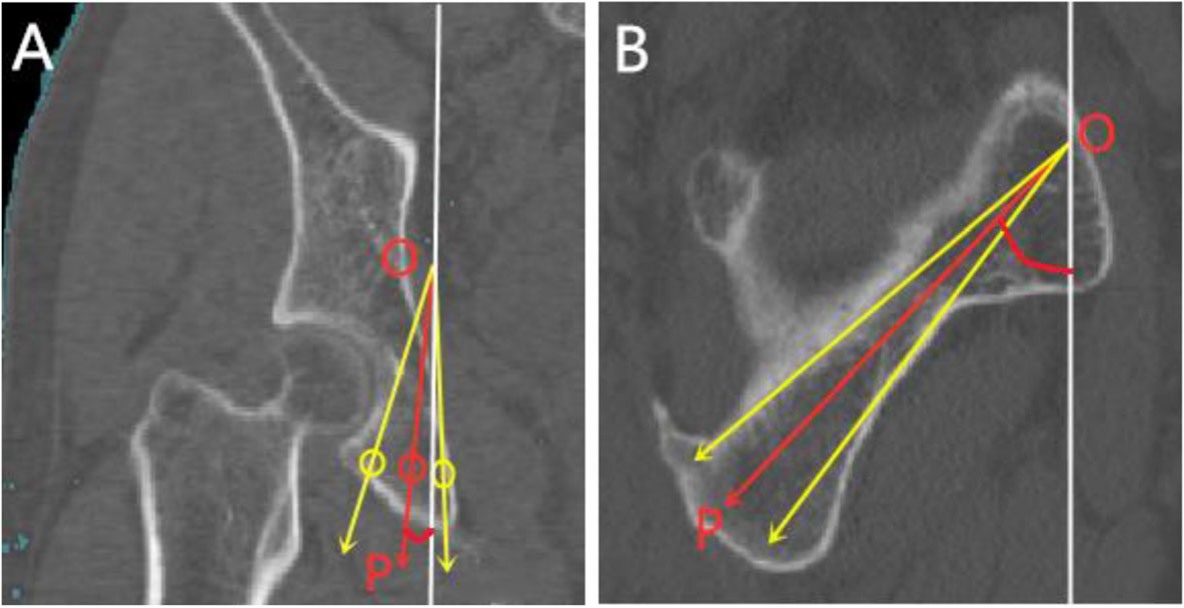
Measurement of screw angle. 5A: Coronal image of pelvis and the white solid line represents the sagittal plane. 5B: Sagittal image of pelvis and white solid line represents coronal plane. The red arrow represents the optimum direction of screw, and the yellow arrows on both sides represent the critical directions

The angles between the virtual screw and the coronal and sagittal planes of the pelvis were measured in the standard anatomical position, and the optimum screw directions were recorded. When all-in screw model was chosen, the point O was fixed, and point P was mobilizable, however the screw could not penetrate the cortex in any direction. We measured the angle between the virtual screw and the coronal and sagittal planes in the critical state to achieve the maximum safety range. In the same way, the maximum safety range of in-out-in screw were gained. We assumed that the angle was positive when the screw was oriented laterally in the sagittal plane, correspondingly the angle was negative when the screw was oriented medially (Fig. 5).

### Statistical analysis

All analyses were performed using SPSS software 23.0(SPSS Inc., Chicago, Illinois). The Shapiro-Wilk test was used to determine whether the data obeyed a normal distribution. The differences of measurement data were compared with independent sample t test. The rank sum test was used on the difference in the composition ratio of ranked data. Simple linear regression analysis was applied to analyze the relevance between the IACD and the MTMAW. The threshold for statistical significance was set at p smaller than 0.05.

## Results

### Sex-specific differences in IACD and the relationship between the IACD and the MTMAW

The IACD of males was 6.15 ± 1.24 mm(4.09 ~ 8.36 mm) and that of females was 5.42 ± 1.01 mm(3.72 ~ 7.62 mm). According to statistical analysis, there was a significant difference between male and female on IACD (t = 3.174, *P* = 0.002). In 71 of 100 pelvises (71%), IACD was ≥5 mm, including 38 males and 33 females; In 29 of 100 pelvises, IACD was < 5 mm, including 12 males and 17 females. The difference in the composition ratio of IACD between men and women was statistically significant(Z = -2.416, *P* = 0.014) (Table 1).

**Table 1:** Comparison of the composition ratio of the IACD between male and female

	Number	IACD (mm) and ratio					
		3–4	4–5	5–6	6–7	7–8	8–9
**Male**	50	0	12(24%)	11(22%)	11(22%)	13(26%)	3(6%)
**Female**	50	2(4%)	15(30%)	17(34%)	11(22%)	5(10%)	0
**Z value**		−2.461					
***P*** **value**		0.014					

*IACD* infra-acetabular corridor diameter

**Fig. 6:**
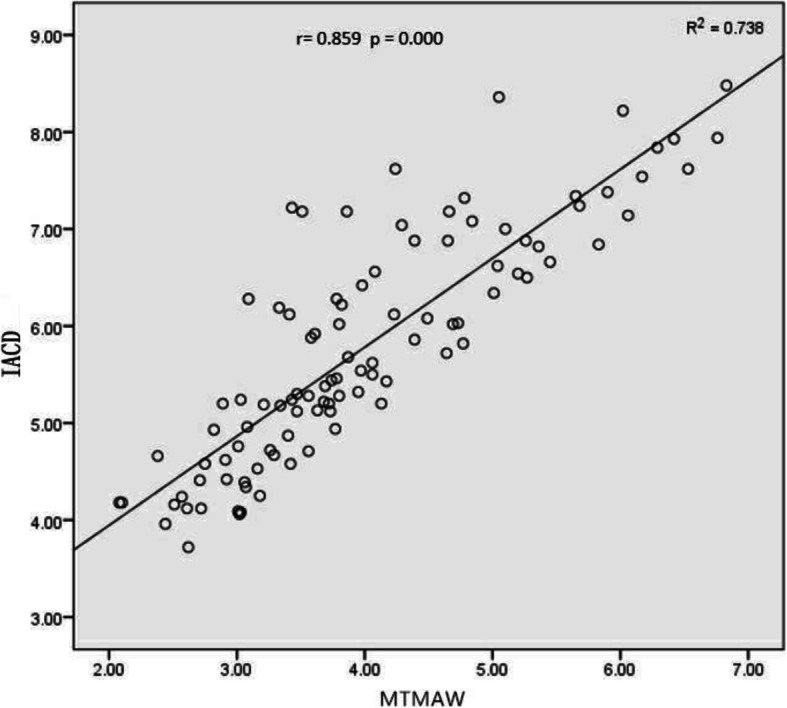
Scatter plot of the correlation between IACD and MTMAW. IACD: infra-acetabular corridor diameter; MTMAW: minimum thickness of medial acetabular wall

From the superior to the inferior of the acetabulum, there were 39 ~ 51 layers with an interval of 1 mm. The MTMAW of males was (4.40 ± 1.23) mm and the distance between the layer of the MTMAW and superior of the acetabulum was (22.80 ± 4.13)mm. As for females, those two parameters were (3.60 ± 0.81) mm and (20.66 ± 3.44) mm respectively. These differences were statistically significant (t = 3.811, *P* < 0.05; t = 2.812, *P* < 0.05). The layer of the MTMAW was roughly located at the center of horseshoe-shaped fovea. There was a positive correlation between IACD and MTMAW(*r* = 0.859)(Fig. 6). The regression equation was IACD = 2.111 + 0.917MTMAW (*R*^2^ = 0.735, *P* < 0.01), the difference was statistically significant.

### Sex-specific differences in all-in screw entry point position and corridor length

The entry point was located at posteromedial of the apex of iliopubic eminence, and the posterior distance and medial distance were (8.03 ± 2.01) mm and (8.49 ± 2.68) mm respectively in males. As for females, those were (8.68 ± 2.35) mm and (8.87 ± 2.79) mm respectively. These differences did not reach statistical significance (*p* > 0.05). The corridor length and distance between entry point and true pelvic rim were significantly longer in males (99.43 ± 4.04 vs 88.83 ± 3.71 mm and 6.61 ± 1.83 vs 4.47 ± 1.41 mm, respectively) (Table 2).

**Table 2:** Comparison of all-in and in-out-in screw parameters between male and female

	Male	Female	t value	*P* value
AB (mm)	8.03 ± 2.01	8.68 ± 2.35	−1.255	0.214
OB (mm)	8.49 ± 2.68	8.87 ± 2.79	−0.593	0.555
OM (mm)	6.61 ± 1.83	4.47 ± 1.41	5.852	**< 0.001**
OP (mm)	99.43 ± 4.04	88.83 ± 3.71	11.440	**< 0.001**
A_1_B_1_ (mm)	10.49 ± 2.58	10.10 ± 2.63	0.395	0.696
O_1_B_1_ (mm)	6.17 ± 1.84	6.63 ± 1.49	−0.755	0.457
O_1_M_1_(mm)	4.22 ± 1.11	3.53 ± 1.17	1.594	0.121
O_1_P_1_ (mm)	98.19 ± 4.37	87.31 ± 4.48	6.496	< 0.001
O_1_D (mm)	20.03 ± 4.00	19.08 ± 3.41	0.688	0.497
O_1_E (mm)	30.64 ± 4.62	25.79 ± 3.34	3.283	0.003
DE (mm)	10.61 ± 2.04	6.71 ± 2.22	4.796	< 0.001

AB: posterior distance between the entry point of all-in screw and the apex of iliopubic eminence; OB: medial distance between the entry point of all-in screw and the apex of iliopubic eminence; OM: distance between entry point of all-in screw and pelvic rim; OP: length of the infra-acetabular corridor (IAC); A1B1: posterior distance between the entry point of in-out-in screw and the apex of iliopubic eminence; O1B1: medial distance between the entry point of in-out-in screw and the apex of iliopubic eminence; O1M1: distance between entry point of in-out-in screw and true pelvic rim; O1P1: length of the infra-acetabular corridor (IAC); O1D: distance between entry point of in-out-in screw and the proximal perforation point in the quadrilateral plate; O1E: distance between entry point of in-out-in screw and the distal perforation point in the quadrilateral plate; DE: length of screw outside of the cortex.

### Sex-specific differences in in-out-in screw entry point position, corridor length and perforation in the quadrilateral plate

The entry point was located at posteromedial of the apex of iliopubic eminence, and the posterior distance and medial distance were (10.49 ± 2.58) mm and (6.17 ± 1.84) mm respectively in males. As for females, those were (10.10 ± 2.63) mm and (6.63 ± 1.49) mm respectively. These differences did not reach statistical significance(*p* > 0.05). Differences of distance between entry point and true pelvic rim and distance between entry point and the proximal perforation in the quadrilateral plate did not reach statistical significance either. The corridor length, distance between entry point and the distal perforation in the quadrilateral plate and length of screw outside of the cortex were significantly longer in males(98.19 ± 4.37 vs 87.31 ± 4.48 mm, 30.64 ± 4.62 vs 25.79 ± 3.34 mm and 10.61 ± 2.04 vs 6.71 ± 2.22 mm, respectively) (Table 2).

### Comparison of optimum screw angle

There was no statistically significant difference in the angle between screw and coronal plane between genders in both groups, however, the difference in angle between screw and sagittal plane had statistical significance. There was no statistical significance on the difference in optimum screw angle between groups in same gender (Table 3).

**Table 3:** of optimum screw angle and screw safety range

	Male	Female	t value	***P*** value
Angle between screw and sagittal plane(°)				
All-in screw	−1.02 ± 6.31	8.19 ± 6.81	−5.912	< 0.001
In-out-in screw	1.48 ± 6.97	7.90 ± 5.45	−2.779	0.010
**t value**	−1.173	0.153		
***P*** **value**	0.246	0.879		
Angle between screw and coronal plane(°)				
All-in screw	53.77 ± 7.64	54.20 ± 7.31	−0.238	0.813
In-out-in screw	55.85 ± 6.37	53.15 ± 7.89	0.979	0.336
**t value**	−0.850	0.467		
***P*** **value**	0.400	0.642		
Safety range of angle between screw and sagittal plane(°)				
All-in screw	7.57 ± 3.57	5.06 ± 2.89	3.220	0.002
In-out-in screw	6.34 ± 1.47	4.60 ± 1.58	2.993	0.006
**t value**	1.709	0.731		
***P*** **value**	0.094	0.468		
Safety range of angle between screw and coronal plane(°)				
All-in screw	8.49 ± 2.37	6.73 ± 2.00	3.347	0.001
In-out-in screw	8.02 ± 3.06	5.89 ± 2.09	2.229	0.034
**t value**	0.547	1.373		
***P*** **value**	0.587	0.176		

### Comparison of screw safety range

The screw safety angle range was wider in male in both groups, however, there was no statistical significance on the difference in safety range between groups in same gender (Table 3).

## Discussion

Open reduction and internal fixation of acetabular fractures provide the most consistent functional results when an anatomic restoration and a congruent articular surface can be achieved [10]. Infra-acetabular screw firstly mentioned by Culemann [3] strengthens the fixation dramatically by constituting periacetabular fixation frame with both osseous columns, the ilioinguinal plate, and supra-acetabular screw fixation. In several biomechanical analysis studies [11, 12], some authors found that additional placement of an infra-acetabular screw significantly increased the fracture fixation strength and reduced the displacement of the fracture. However, compared with the acetabular anterior and posterior corridors, the infra-acetabular corridor is significantly narrower, which may be even more significant for Asians. Additionally, infra-acetabular corridor is surrounded by obturator nerves and vessels [13], which increases the risk of iatrogenic injury when screw is inserted. Therefore, it is essential to conduct a detailed study on the placement method and anatomical parameters of the infra-acetabular screw.

In the original article by Gras et al. [6], they found the mean IACD of males and females were 7.7 mm and 6.9 mm respectively, the corridor with a diameter of at least 5 mm existed in 93% of cases (90% in females versus 94% in males) and the mean corridor length of males and females were 106.4 mm and 96.2 mm. However, in our study, the mean IACD of males and females were (5.15 ± 1.25) mm and (4.42 ± 1.01) mm respectively, the corridor with a diameter of at least 5 mm existed in 71% of cases (66% in females versus 76% in males), and the mean corridor length of males and females in all-in screw group were (99.43 ± 4.04) mm and (88.83 ± 3.71) mm and those data in in-out-in screw group were (98.19 ± 4.37) mm and (87.31 ± 4.48)mm. Obviously, the diameter and length of infra-acetabular corridor studied by Gras et al. are greater than the results in our study, which may be caused by the racial difference. The infra-acetabular corridor is narrow and long and the safety range is limited. Therefore, tiny angular deviation may cause iatrogenic damage to the peripheral neurovascular bundles and hip joint [14]. Cai XH et al. [15] have shown that when 1/3 ~ 1/2 of the screw diameter is located in the bone of the quadrilateral plate, it can effectively resist the separation of fracture fragment, provide rigid fixation and avoid the screw from penetrating the joint. In our study, we found that the minimum ICAD of males and females were respectively 4.09 mm and 3.72 mm, and it was difficult to insert 3.5 mm screws in this situation. Whereupon we proposed that the screw placement method can be selected according to IACD. When IACD was ≥5 mm, the 3.5 mm all-in screw was selected; When IACD was <5 mm, we inserted the 3.5 mm in-out-in screw and made 1/2 of the screw diameter exposed out of the quadrilateral plate cortex. Arlt et al. [16] found that the infra-acetabular corridor showed a double-cone shape with the isthmus located in the region of the acetabular fovea as the limiting anatomical structure. Additionally, the body weight, body height, and the diameter of Köhler’s teardrop in the anteroposterior X-ray view showed significant positive correlations with the corridor volume. In our study, IACD had a positive correlation with MTMAW. Hence, based on the regression equation, we can calculate the IACD by the MTMAW which can be measured by CT scan, thereby the screw placement method can be selected preoperatively. When the MTMAW is greater than 3.15 mm, all-in screw can be inserted.

**Fig. 7:**
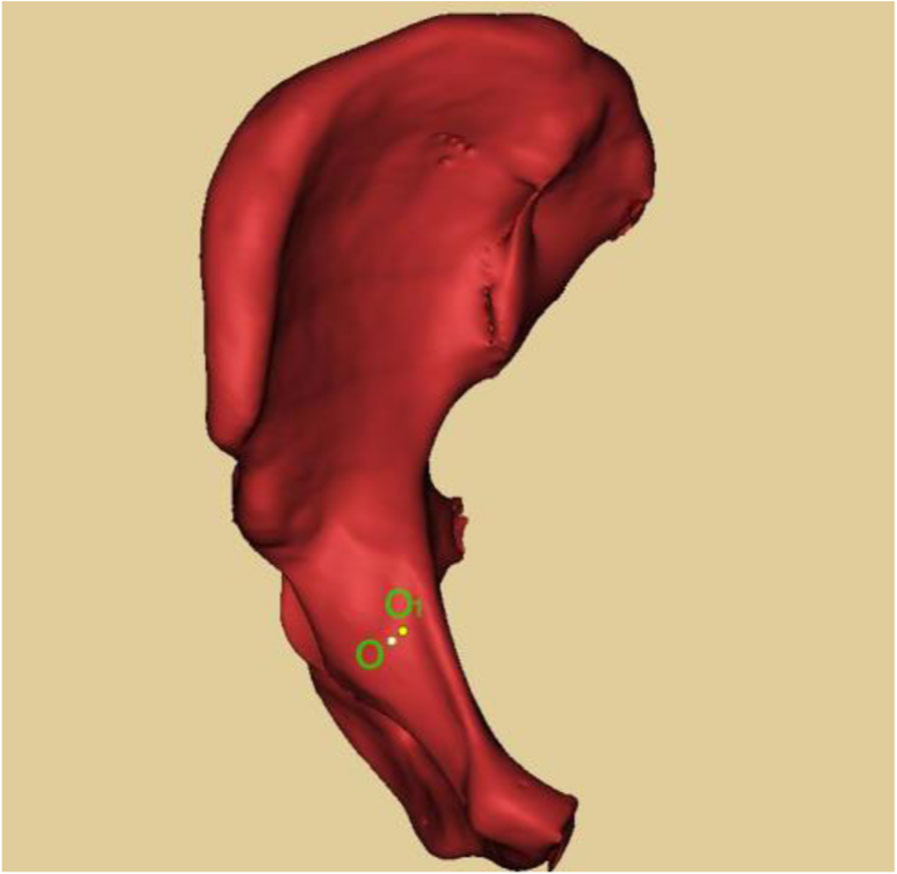
Comparison of entry point between all-in screw and in-out-in screw. Point O: Entry point of all-in screw. Point O_1_: Entry point of in-out-in screw

Culemann et al. [3] described that the entry point for the infra-acetabular screw is 1 cm caudal of the iliopectineal eminence in the mid-width of the pubic ramus. Gras et al. [4] measured the distance between anterior superior iliac spine and pubic symphysis and the distance between pubic symphysis and screw entry point, they found the mean ratio of these two distances is 1.36. Whereas these two methods are not utility in clinical practice. Baumann [17] found that the entry point is located at the posteromedial of apex of iliopubic eminence. The relationship between the entry point and the iliopubic eminence has no relevance with gender, age, or body type. In our study, the apex of iliopubic eminence and true pelvic rim were regarded as the reference point to measure the entry point, which could be confirmed by palpation [18] and was convenient to locate the entry point during the operation. We found that the all-in screw entry point was closer to the apex of iliopubic eminence than the results in the study by Baumann, which may be caused by the racial differences. The all-in screw exit point was roughly located at the medial of the middle ischial tuberosity. The infra-acetabular corridor was surrounding by obturator nerves and vessels, the in-out-in screw entry point was located at lateral of the obturator groove in order to avoid iatrogenic injury. Compared with all-in screws, the in-out-in screw entry point was round 2 mm outwards and backwards, and closer to true pelvic rim (Fig. 7). When in-out-in screw is applied, the periosteum dissector can be used to push away the obturator nerves and vessels to protect them. 90% of length of in-out-in screw in the sclerotin can ensure the stability the screw fixation.

According to the data in our study, there is no statistical significance on the difference of optimum screw angle between groups in same gender. The angle between screw and the sagittal plane was more medial tilted in males than that in females in both groups, which may be caused by the morphological differences of pelvis in genders. The pelvis is “funnel-shaped” in males, however that is “barrel-shaped” in females. The infra-acetabular corridor was nearly parallel to the sagittal plane and the medial inclination angle was round (0.42 ± 6.49) ° in males. However, the angle was lateral inclined in females, which was round (8.09 ± 6.33) °. Additionally, the screw was (54.06 ± 7.37) ° posterior inclination with the coronal plane in both genders.

**Fig. 8:**
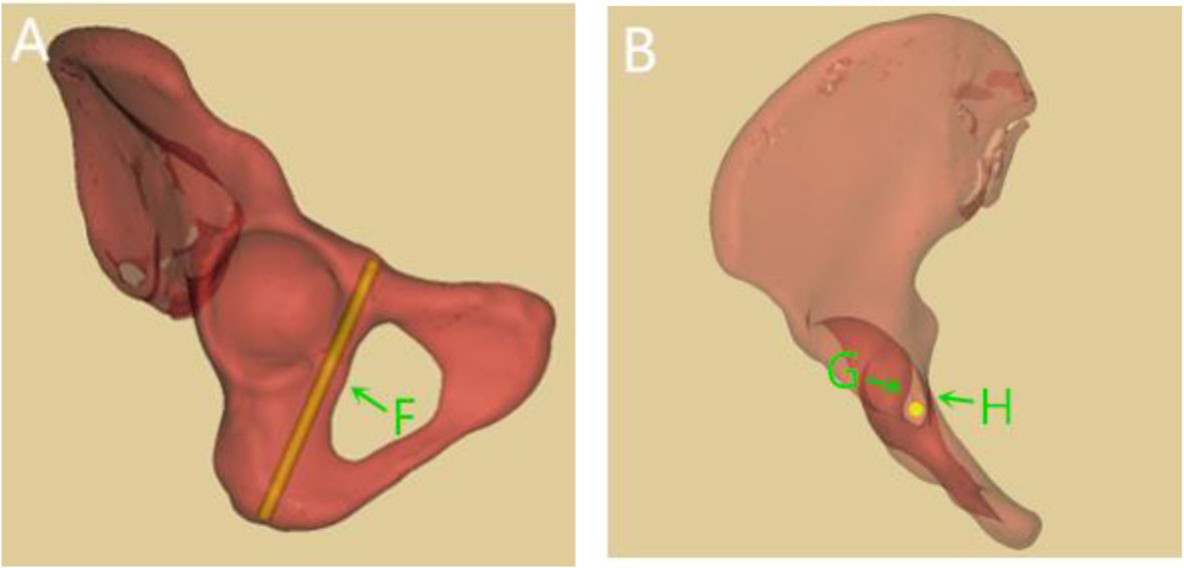
Perspective position. **A** Obturator oblique and outlet view; F: Posterior of obturator. **B** Axial perspective view; G: Acetabulum; H: Quadrilateral plate

The inlet, outlet, iliac oblique and obturator oblique views are commonly used to verify whether the screw perforates the joint or cortex during the process of screw placement [19, 20]. However, the anatomy peri infra-acetabular corridor is quite complicated, and the above-mentioned fluoroscopic methods cannot fully assess whether the screw penetrates the joint, the quadrilateral plate and the posterior of obturator. If the screw posterior inclination is too small, it is easy for the screw to penetrate the posterior of obturator and damage the obturator nerve and vascular bundles. The infra-acetabular corridor is narrow and long and the safety range is limited. If the angle between the screw and the sagittal plane is too large during the screw placement process, there is a risk of penetrating the acetabular joint. In the original research by Culemann et al. [3], c-arm rotated to the injured side and tilted 30°caudally in the obturator oblique and outlet views with patient supine for the control of correct screw path (Fig. 8A). In our study, we trend to rotate the c-arm to the injured side and tilted 50°-55° cranially. The infra-acetabular screw in males is nearly parallel to the sagittal plane, however it is slightly lateral tilted in females. According to the orientation of screw, we adjust the position of c-arm to eliminate the angle between the screw and the coronal and sagittal planes. In this way, the screw becomes a dot in the perspective view. We can verify whether the screw penetrate the joint or quadrilateral plate through this view (Fig. 8B).

The infra-acetabular corridor is quite narrow, which makes a challenge for the orthopedists to insert the screw. Hence, Gras et al. [21] recommended inserting the infra-acetabular screw under the guidance of 3D navigation system. Lehmann et al. [18] designed a new electromagnetic navigation system and completed infra-acetabular screw placement for 22 of 24 patients with the help of this new system. However, these technologies are demanding for the hospital facilities, which makes a difficulty to widely apply around the world, especially in the developing countries. Therefore, if a portable infra-acetabular screw sighting device could be designed based on the results in this study, the safety and effectiveness of the operation will be improved.

Some limitations of our study must be noted. Firstly, the selection of the screw placement method and the measurement of the screw placement parameters are based on the three-dimensional reconstructed pelvic model, and its effectiveness needs further clinical verification. Secondly, whether there is a difference in the fixation strength of the all-in and in-out-in screws requires biomechanical analysis. Thirdly, further research can also verify the application value of the regression equation. In addition, the participators in our study may not be sufficient to represent the entire population, and the different races may have different results.

In conclusion, 27% of the ICAD were less than 5 mm in our study, which means that around 1/4 of the patients could not be inserted in all-in screws. Preoperative measurement of MTMAW based on CT can determine the method of screw placement. Compared with all-in screw, the in-out-in screw entry point was around 2 mm outwards and backwards, and closer to true pelvic rim. The diameter of ICAD in women is narrower than that in men, and the angle between screw and the sagittal plane was more lateral tilted in females.

## Data Availability

The datasets generated and analyzed during the current study are not publicly available due to individual privacy of participants but are available from the corresponding author on reasonable request.

## Abbreviations

ICAD: Infra-acetabular corridor diameter
MTMAW: Minimum thickness of medial acetabular wall
CT: Computed tomography
3D: Three-dimensional
IAS: Infra-acetabular screw
IAC: Infra-acetabular corridor
DICOM: Digital imaging communication in medicine
STL: Stereolithography
